# Oxytocin modulates hippocampal perfusion in people at clinical high risk for psychosis

**DOI:** 10.1038/s41386-018-0311-6

**Published:** 2019-01-09

**Authors:** Cathy Davies, Yannis Paloyelis, Grazia Rutigliano, Marco Cappucciati, Andrea De Micheli, Valentina Ramella-Cravaro, Umberto Provenzani, Mathilde Antoniades, Gemma Modinos, Dominic Oliver, Daniel Stahl, Silvia Murguia, Fernando Zelaya, Paul Allen, Sukhi Shergill, Paul Morrison, Steve Williams, David Taylor, Philip McGuire, Paolo Fusar-Poli

**Affiliations:** 1 Early Psychosis: Interventions & Clinical-detection (EPIC) Lab, Department of Psychosis Studies, Institute of Psychiatry, Psychology & Neuroscience, King’s College London, London, UK; 2 Department of Neuroimaging, Institute of Psychiatry, Psychology & Neuroscience, King’s College London, London, UK; 3 National Institute for Health Research (NIHR) Biomedical Research Centre (BRC), South London and Maudsley NHS Foundation Trust, London, UK; 4 Department of Brain and Behavioral Sciences, University of Pavia, Pavia, Italy; 5 Department of Psychosis Studies, Institute of Psychiatry, Psychology & Neuroscience, King’s College London, London, UK; 6 Department of Biostatistics and Health Informatics, Institute of Psychiatry, Psychology & Neuroscience, King’s College London, London, UK; 7 Tower Hamlets Early Detection Service (THEDS), East London NHS Foundation Trust, London, UK; 8 Department of Psychology, University of Roehampton, London, UK; 9 Institute of Pharmaceutical Science, King’s College London, London, UK; 10 Outreach And Support in South London (OASIS) Service, South London and Maudsley NHS Foundation Trust, London, UK

**Keywords:** Neurophysiology, Social neuroscience, Psychosis, Translational research

## Abstract

Preclinical and human studies suggest that hippocampal dysfunction is a key factor in the onset of psychosis. People at Clinical High Risk for psychosis (CHR-P) present with a clinical syndrome that can include social withdrawal and have a 20–35% risk of developing psychosis in the next 2 years. Recent research shows that resting hippocampal blood flow is altered in CHR-P individuals and predicts adverse clinical outcomes, such as non-remission/transition to frank psychosis. Previous work in healthy males indicates that a single dose of intranasal oxytocin has positive effects on social function and marked effects on resting hippocampal blood flow. The present study examined the effects of intranasal oxytocin on hippocampal blood flow in CHR-P individuals. In a double-blind, placebo-controlled, crossover design, 30 CHR-P males were studied using pseudo-continuous Arterial Spin Labelling on 2 occasions, once after 40IU intranasal oxytocin and once after placebo. The effects of oxytocin on left hippocampal blood flow were examined in a region-of-interest analysis of data acquired at 22–28 and at 30–36 minutes post-intranasal administration. Relative to placebo, administration of oxytocin was associated with increased hippocampal blood flow at both time points (*p* = .0056; *p* = .034), although the effect at the second did not survive adjustment for the effect of global blood flow. These data indicate that oxytocin can modulate hippocampal function in CHR-P individuals and therefore merits further investigation as a candidate novel treatment for this group.

## Introduction

At present, there is a lack of effective treatments for individuals at Clinical High Risk of Psychosis (CHR-P [1]). Recent studies suggest that existing interventions do not significantly impact on transition to psychosis [2], attenuated positive [3] or negative symptoms [4], or social and functional outcomes [5]. Novel treatments for this population are therefore needed [6].

**Fig. 1:**
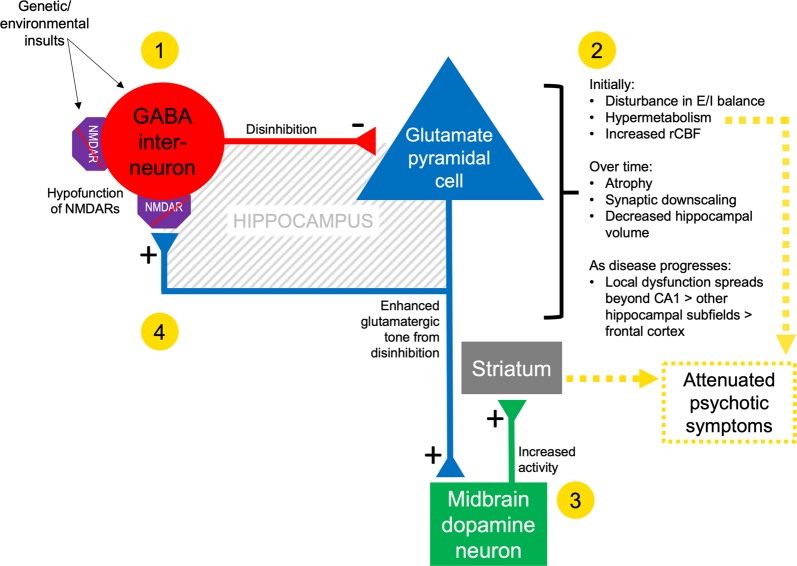
Simplified schematic of proposed neural circuit mechanisms of hippocampal pathophysiology in those at CHR-P. In (**1**), low glutamate signal/input from hypofunctioning NMDARs (akin to ‘faulty homeostatic sensors’) leads GABAergic interneurons to seek to homeostatically increase excitation by reducing inhibition (disinhibition) of glutamatergic pyramidal cells. However, by disinhibiting pyramidal cells (and thus increasing glutamate signalling) in this dysfunctional neural environment, the potential homeostatic adaptation becomes allostatic (**2**). In (**3**), enhanced excitation leads to an overdrive in the responsivity of midbrain dopamine neurons which project to the associative striatum (note that the connection between hippocampal pyramidal cells and midbrain dopamine neurons is presented as monosynaptic but is actually polysynaptic via the ventral striatum and ventral pallidum). Completing the (simplified) circuit, local glutamatergic tone is increased in (**4**) but is not detected as such by hypofunctioning NMDARs on GABAergic interneurons. For detailed original diagrams and discussion of evidence for this proposed circuit or its component processes, see [7, 11, 12, 14, 74]. *Glu* glutamate, *NMDAR* N-methyl-D-aspartate receptor, *E/I* excitation/inhibition

A substantial body of scientific work places aberrant hippocampal structure and function at the core of neurobiological mechanisms underlying the onset of psychosis [7]. Evidence from post-mortem, neuroimaging and preclinical research suggests that the onset of attenuated psychotic symptoms may be driven by dysregulated glutamate neurotransmission in the Cornu Ammonis 1 (CA1) region of the hippocampus, which is thought to lead to hypermetabolism and altered (increased) blood flow [7–10]. Enhanced glutamatergic tone in CA1 induces allostatic adaptations [11] in γ-aminobutyric acid (GABA)-ergic neurotransmission, with consequent disinhibition of pyramidal neurons (Fig. 1) [12]. These changes may lead to disturbed neural excitation/inhibition balance, and via polysynaptic projection pathways to the midbrain/striatum, to midbrain hyperdopaminergia [13, 14]. As the CHR-P state progresses to the first episode of psychosis, the functional perturbations once localised to (particularly the left) CA1 may spread to extra-hippocampal regions such as the frontal cortex [7, 8], and excitotoxic as well as atrophic processes culminate in hippocampal volume loss—structural changes—beginning in CA1 [15–17]. These findings are consistent with evidence that CHR-P individuals show increased resting regional cerebral blood flow (rCBF) in the hippocampus relative to controls [18, 19], and normalisation (reduction) of left hippocampal rCBF is associated with remission from the CHR-P state [18]. Hippocampal rCBF in CHR-P individuals has also been correlated with cortical GABA levels [20].

The neuropeptide oxytocin is a key modulator of social, sexual and emotional processes [21], including hypothalamic-pituitary-adrenal axis regulation [22], emotion recognition [23], social memory [24] and reward, and possesses anxiolytic [21, 22] and prosocial properties [25, 26]. Previous work in healthy males [27] suggests that an acute dose of intranasal oxytocin significantly increases rCBF in limbic and midbrain regions, including the hippocampus. Preclinical studies have also identified the hippocampus as a key target for oxytocin-mediated effects, with oxytocin enhancing the signal-to-noise ratio of CA1 pyramidal cell firing by selectively targeting GABAergic interneuron function [28, 29]—part of the neural circuit implicated in psychosis onset [6, 12, 14]. Oxytocin is further linked to these mechanisms via its role in neural circuit maturation in the pre- and peri-natal period, responsible for the excitatory-to-inhibitory switching of GABAergic signalling [30] and controlling dendrite complexity [31], synapse density [31] and the onset of synchronous firing in developing hippocampal pyramidal neurons [32]. Preclinical research also indicates that oxytocin can protect hippocampal CA1 plasticity and memory from the effects of stress [33], which is also implicated in the onset of psychosis [13].

The aim of the present study was to examine the acute effects of oxytocin on hippocampal rCBF in CHR-P individuals. We focused on the left (and not right) hippocampus because previous CHR-P research has repeatedly implicated the left hippocampus in the pathophysiology of psychosis risk [7, 8, 10, 18, 20, 34, 35], with left (and not right) hippocampal rCBF associated with clinical outcomes [18]. In addition, previous oxytocin research has reported distinctly left-lateralised effects of oxytocin on cerebral blood flow [27]. In view of evidence that certain subregions of the hippocampus may be particularly involved in the risk for psychosis, and may be particularly influenced by oxytocin, we also investigated whether the effects of oxytocin were specific to different hippocampal subregions. Finally, we explored whether oxytocin had additional effects outside the hippocampal region in a whole-brain analysis. The a priori evidence for altered hippocampal rCBF in CHR-P individuals vs controls comes from the same clinical sample as the current study—recruited from the Outreach And Support in South London (OASIS) service [36]—and has already been replicated in a further sample recruited from this clinic [18, 19]. Leveraging these findings, the current study adopted a within-subject crossover design. Our first hypothesis was that oxytocin would modulate hippocampal rCBF in CHR-P subjects. We did not hypothesise a specific effect direction because the aim of this study was to demonstrate disease-target (hippocampal) engagement. Secondary predictions were that its effects would be particularly evident in the CA1 subregion, and that it would also influence rCBF outside the hippocampus in regions implicated in social and emotional processing.

## Patients & methods

### Participants

The study received National Research Ethics Service approval (14/LO/1692) and all subjects gave written informed consent. Thirty male, help-seeking CHR-P individuals aged 18–35 were recruited from two specialist early detection services—the OASIS [36] and Tower Hamlets Early Detection Service (THEDS). A CHR-P status was determined using the Comprehensive Assessment of At-Risk Mental States (CAARMS) 12/2006 criteria [37]. Briefly, subjects met one or more of the following subgroup criteria: (a) attenuated psychotic symptoms, (b) brief limited intermittent psychotic symptoms (BLIPS, psychotic episode lasting < 1 week, remitting without treatment), or (c) either schizotypal personality disorder or first-degree relative with psychosis [37], all coupled with functional decline. Individuals were excluded if there was a history of previous psychotic disorder (with the exception of BLIPS, some of whom may meet acute and transient psychotic disorder criteria [38]) or manic episode, exposure to antipsychotics, neurological disorder or current substance-use disorder, estimated IQ < 70, acute intoxication on the day of scanning, and any contraindications to magnetic resonance imaging (MRI) or intranasal oxytocin or placebo. History of Axis I disorder(s) was not an exclusion criterion due to the transdiagnostic nature of the CHR-P state and the high prevalence of such diagnoses within these populations [39].

### Design, materials, procedure

We used a randomised, double-blind, 40 IU intranasal oxytocin vs placebo single-dose challenge in a crossover design (1-week wash out). During each challenge, subjects underwent an MRI scan which started at 1130 h to minimise potential effects of diurnal variation in oxytocin or vasopressin [27]. Anxiety was measured using the State-Trait Anxiety Inventory (STAI) prior to each scan (and prior to intranasal administration) so that pre-scan anxiety score could be included in statistical models as a covariate. For descriptive purposes, we also collected information on medication history, use of alcohol (Alcohol Use Disorders Identification Test), tobacco and cannabis, functioning using the Global Functioning Role and Social scales [40] and later transition status. Intranasal administration followed recommended guidelines and a protocol adopted by a previous study conducted at our institute [27]. Briefly, participants self-administered one puff (4 IU) of intranasal oxytocin or matched placebo every 30 s, alternating between nostrils, until 40 IU had been administered (Supplementary Materials and Methods). During the scan, participants were asked to maintain their gaze on a centrally-placed fixation cross.

### MRI acquisition and image processing

All scans were conducted on a General Electric Discovery MR750 3 Tesla system (General Electric, Chicago, USA) using a 32-channel head coil. Measurement of Cerebral Blood Flow (CBF) was carried out using a 3D pseudo-continuous Arterial Spin Labelling (3D-pCASL) sequence during two consecutive runs: 22–28 (run 1) and 30–36 (run 2) min post-intranasal administration. The timing of the two runs was selected based on previous findings of the spatiotemporal profile of oxytocin-induced cerebral blood flow changes in healthy males, which demonstrated sustained effects over a ~20–73 min period (post-intranasal administration) [27]. For each subject, we also computed a mean (average) CBF map from the CBF maps for runs 1 and 2. ASL data were preprocessed using the Automatic Software for ASL Processing (ASAP) 2.0 toolbox [41] running in Statistical Parametric Mapping version 12 (SPM12; https://www.fil.ion.ucl.ac.uk/spm/) on Matlab R2017a. 3D-pCASL acquisition parameters and image preprocessing procedures were conducted in line with previous studies and are detailed in the Supplementary Materials and Methods.

### Statistical analysis

Statistical analyses were performed in STATA SE14.2.

### Pre-scan anxiety scores

For pre-scan anxiety (STAI) scores, missing data were imputed using next-observation-carried-backward (Supplementary Materials and Methods). Differences in pre-scan anxiety scores in the oxytocin vs placebo conditions was assessed using a paired t-test. In line with previous CHR-P studies [18, 19] and because anxiety has been demonstrated to have systematic effects on CBF [42] (including rCBF specifically in the hippocampus [43]), all analyses included mean-centred pre-scan anxiety as a covariate.

### Global cerebral blood flow (CBF)

To measure global CBF signal, we used the ASAP toolbox to extract average CBF values from a grey matter mask for each subject. The ICBM-152 mask was obtained from the DARTEL toolbox in SPM and thresholded to contain voxels with a >.25 probability of being grey matter. To ascertain whether global CBF was significantly different in the oxytocin relative to placebo condition, we conducted repeated-measures analyses of covariance (RM-ANCOVA) in STATA for run 1, run 2, and the mean of the runs (separately), using pre-scan anxiety as covariate (Supplementary Materials and Methods). All subsequent analyses were conducted with and without global CBF as covariate.

### Hippocampal ROI rCBF

**Fig. 2:**
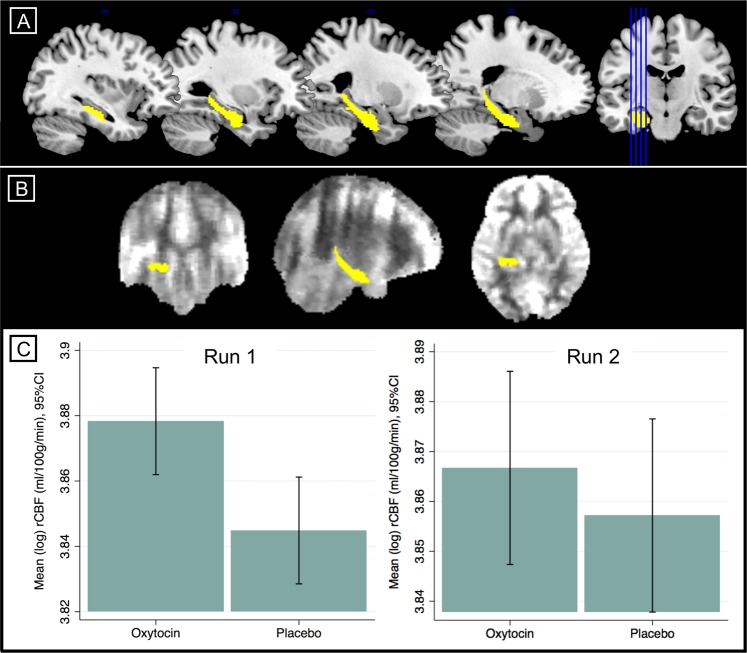
rCBF Effects in Left Hippocampus. **a** ROI mask for the left hippocampus (yellow) overlaid on a standard brain template, and (**b**) overlaid on a representative subject-level cerebral blood flow map in normalised space, and (**c**) bar charts showing mean hippocampal rCBF in the oxytocin and placebo conditions in run 1 and run 2 after adjustment for global effects

Effects of oxytocin on hippocampal rCBF were determined using a region-of-interest (ROI) approach. A left hippocampal ROI was defined anatomically in MNI space using the cytoarchitectonic probabilistic atlas [44] as implemented in the Anatomy toolbox [45] in SPM (Figs. 2a, b). The ROI mask was composed of regions CA1, CA2, CA3, dentate gyrus, and subiculum. Mean rCBF values for the ROI were extracted for each subject using ASAP toolbox and entered into RM-ANCOVAs in STATA (Supplementary Materials and Methods). Our primary analyses tested for effects in each of the two runs separately. However, due to the low signal-to-noise ratio inherent in ASL data, we also conducted an analysis of the mean effect across the two runs, which can help to reduce noise if the effects are stable [27]. We contained the family-wise error (FWE) rate at α = .05 using the Hochberg procedure, which is a 'sharper' and more powerful version of the Bonferroni adjustment and which allows non-independence between statistical tests [46]. Original *p* values (two-tailed) are reported alongside indication of Hochberg correction survival (i.e., whether or not they remain significant (survive) after accounting for FWE). Effect sizes are reported as omega-squared (*ω*^2^).

### Exploratory/supplemental analyses

**Fig. 3:**
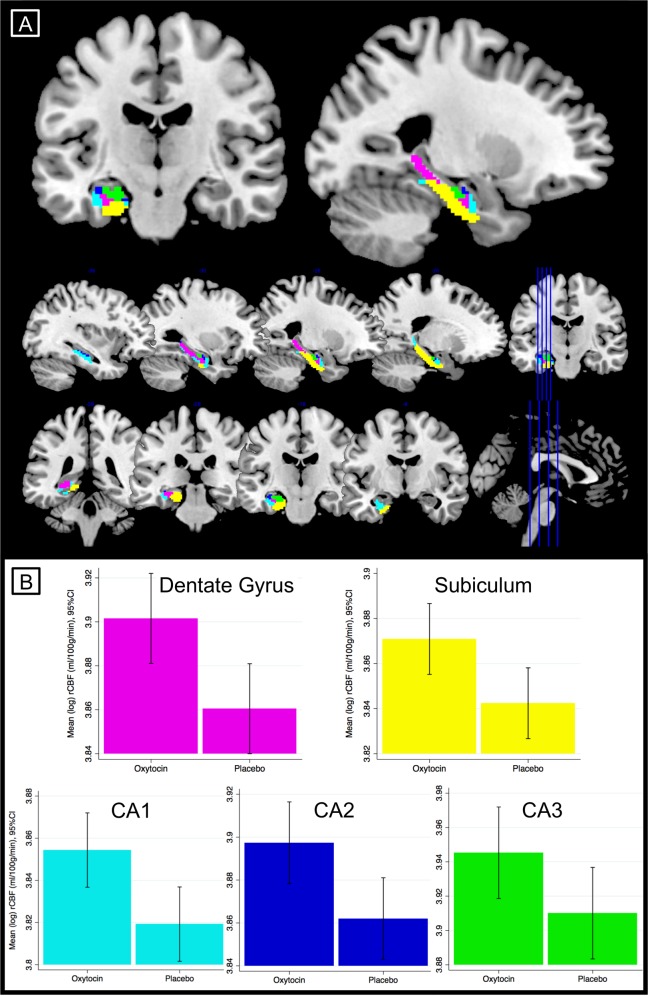
rCBF in Left Hippocampal Subregions. **a** ROI masks for left hippocampal subregions: dentate gyrus (pink), subiculum (yellow), CA1 (cyan), CA2 (blue), and CA3 (green) displayed on a standard brain template, and (**b**) bar charts showing mean hippocampal subregion rCBF in the oxytocin and placebo conditions in run 1 after adjustment for global effects

We used analogous procedures to those described directly above to extract mean rCBF values for each hippocampal subregion, using separate masks for left CA1, CA2, CA3, dentate gyrus, and subiculum (Fig. 3a; and Supplementary Materials and Methods). No multiplicity correction was applied as subregion analyses were exploratory. Finally, for completeness we examined whole-brain effects in runs 1, 2 and the mean of the runs—separately—using paired t-tests (second-level analysis) in SPM, with pre-scan anxiety and with/without global CBF signal as nuisance covariates. We conducted a whole-brain search using cluster level inference (cluster forming threshold: *p* < .005; cluster reported as significant at *p* < .05 using FWE correction in SPM). Analyses were restricted using the explicit ICBM mask again thresholded to contain voxels with >.25 probability of being grey matter.

## Results

### Sample characteristics

Demographic and clinical characteristics of the sample are presented in Table 1. All participants completed the study with no drop-outs. No adverse side effects were clinically observed. One subject was removed due to protocol violations, leaving a sample of *N* = 29. There was a significant difference in pre-scan (pre-intranasal administration) anxiety scores in the oxytocin vs placebo condition (oxytocin [mean ± SE] = 37.4 ± 1.9; placebo = 33.4 ± 1.7; *t*(28) = 2.46, *p* = .020), which may have arisen by chance or due to slightly more individuals receiving treatment order oxytocin > placebo (*N* = 15) vs placebo > oxytocin (*N* = 14).

**Table 1:** Participant demographic and clinical characteristics

	Variable	Total sample (*N* = 30)
Demographic	Age, years; mean (SD)	23.2 (4.7)
	Age range, years	18–35
	Sex, male/female	30/0
	Ethnicity (White/Black/Asian/Mixed)	16/6/4/4
	Handedness, right/left	26/4
	Education, years; mean (SD)	13.2 (1.9)
Clinical	CHR-P Subtype^a^ (BLIPS/APS/GRD)	6/23/1
	CAARMS attenuated positive symptoms^b^; mean (SD)	11.7 (3.3)
	Transition to psychosis (yes/no)^c^	4/26
	Baseline anxiety score^d^; mean (SD)	35.6 (8.7)
	GF social score; mean (SD)	6.8 (1.5)
	GF role score; mean (SD)	7.0 (1.7)
	Current antidepressant medication (yes/no)	8/22
	Current antipsychotic medication (yes/no)	0/30
	Current benzodiazepine medication (yes/no)	1/29
Substance Use	Current smoker (yes/no)	17/13
	Cigarettes/day; mean (SD)	9.8 (6.0)
	Cannabis use^e^; median (range)	2 (0–4)
	Alcohol, AUDIT total; mean (SD)	7.2 (7.7)

^a^Comprehensive Assessment of At-Risk Mental States (CAARMS) subgroup; *BLIPS* brief limited intermittent psychotic symptoms, *APS* attenuated psychotic symptoms, *GRD* genetic risk and deterioration

^b^Sum of the global (severity) ratings for positive subscale items (P1-P4) of the CAARMS

^c^The 4 transitions occurred within 26 months but the follow up is still ongoing

^d^Mean of pre-scan anxiety scores across conditions as measured by the State Trait Anxiety Inventory (STAI)

^e^Cannabis use: 0 = never, 1 = experimental use (tried occasionally), 2 = occasional use (small quantities from time to time), 3 = moderate use (moderate quantities regularly / large amounts occasionally), 4 = severe use (frequently used large quantities, often to intoxication/debilitation). *AUDIT* alcohol use disorders identification test, *CHR-P* clinical high risk for psychosis, *GF* global functioning (role and social) scale

### Global CBF

There was no significant difference in global grey matter CBF values (ml/100 g/min) in the oxytocin relative to the placebo condition in the mean of both runs (oxytocin [marginal mean ± SE] = 52.91 ± 0.91; placebo = 50.23 ± 0.91; *F*(1,27) = 4.00, *p* = .056) or run 1 (oxytocin = 52.89 ± 0.94; placebo = 50.44 ± 0.94; *F*(1,27) = 3.14, *p* = .088), but a significant difference was observed in run 2 (oxytocin = 52.94 ± 0.90; placebo = 50.03 ± 0.90; *F*(1,27) = 4.75, *p* = .038).

### Hippocampal rCBF

rCBF values for all runs were log transformed due to deviations from distributional assumptions for parametric tests. Compared to placebo, oxytocin administration was associated with increased hippocampal rCBF in run 1 (*F*(1,27) = 9.06, *p* = .0056; *ω*^2^ = .223), run 2 (*F*(1,27) = 4.96, *p* = .034; *ω*^2^ = .124) and the mean of the two runs (*F*(1,27) = 7.31, *p* = .012; *ω*^2^ = .184), all of which survived Hochberg multiplicity correction (Figure S1). After controlling for global signal effects, oxytocin administration was associated with increased hippocampal rCBF in run 1 (*F*(1,26) = 7.68, *p* = .010; *ω*^2^ = .198) which survived multiplicity correction (Fig. 2c). The effects were no longer evident in run 2 (*F*(1,26) = 0.44, *p* = .51; Fig. 2c) or in the mean of the runs (*F*(1,26) = 3.27, *p* = .082). Exclusion of participants taking antidepressants (*N* = 8) and benzodiazepines (*N* = 1) in sensitivity analyses made no material change to the unadjusted effects on hippocampal rCBF (Supplementary Materials and Methods).

### Exploratory/supplemental analyses

#### Hippocampal subregions

Hippocampal subregion effects were explored in run 1 only. Relative to placebo, oxytocin administration was associated with increased rCBF in all hippocampal subregions, including CA1 (*F*(1,27) = 9.44, *p* = .0048; *ω*^2^ = .232), CA2 (*F*(1,27) = 9.33, *p* = .0050; *ω*^2^ = .229), CA3 (*F*(1,27) = 6.83, *p* = .014; *ω*^2^ = .172), subiculum (*F*(1,27) = 7.61, *p* = .010; *ω*^2^ = .191) and particularly the dentate gyrus (*F*(1,27) = 10.11, *p* = .0037; *ω*^2^ = .246)(Figure S2). After controlling for global CBF effects, oxytocin administration was associated with increased rCBF in CA1 (*F*(1,26) = 7.29, *p* = .012; *ω*^2^ = .189), CA2 (*F*(1,26) = 6.32, *p* = .018; *ω*^2^ = .165), subiculum (*F*(1,26) = 6.03, *p* = .021; *ω*^2^ = .157) and dentate gyrus (*F*(1,26) = 7.40, *p* = .011; *ω*^2^ = .192), but no difference was found in CA3 (*F*(1,26) = 3.20, *p* = .086)(Fig. 3b). As noted above, these results were not corrected for multiple comparisons.

#### Whole-brain

Since there was no significant difference in global signal, unadjusted whole-brain results (including for the mean of the runs) are reported in Table 2 (see Table S1 and Supplementary Material for global signal-adjusted results). In run 1, oxytocin administration was associated with increased perfusion in a large predominantly left-lateralised cluster spanning the cerebellum, hippocampus, parahippocampal gyrus and visual cortex, with a peak in the cerebellum (*p*_FWE_ < .05). There were no regions where perfusion decreased after oxytocin. In run 2, oxytocin was associated with increased perfusion in a large left-hemisphere cluster spanning the thalamus, parahippocampal gyrus, hippocampus, and fusiform gyrus, with a peak in the parahippocampal gyrus (*p*_FWE_ < .05), and in a separate right-hemisphere cluster with a peak in the superior parietal lobule (*p*_FWE_ < .05).

**Table 2:** Effects of oxytocin vs placebo on whole-brain CBF (without adjustment for global CBF effects)

Cluster Description	Hemisphere	*k*	P_(FWE-corr)_	Peak coordinates			Peak description
				x	y	z	
**Run 1, Oxytocin** **>** **Placebo**							
Left cerebellum, visual cortex, parahippocampal gyrus, hippocampus, fusiform gyrus, lingual gyrus; right cuneus, calcarine gyrus, visual cortex, cerebellum	Left	3904	<.05	−26	−32	−36	Cerebellum (culmen)
				−20	−46	−28	Cerebellum (culmen)
				−24	−72	−2	Lingual gyrus
**Run 1, Placebo** **>** **Oxytocin**							
None							
**Run 2, Oxytocin** **>** **Placebo**							
Left cerebellum, fusiform gyrus, parahippocampal gyrus, hippocampus, lingual gyrus, thalamus; right cerebellum	Left	3117	<.05	−36	−44	−8	Parahippocampal gyrus
				−2	−80	−34	Cerebellum (pyramis)
				−16	−58	−12	Cerebellum (culmen)
Right superior parietal lobule, precuneus, calcarine gyrus, cuneus, visual cortex; left visual cortex	Right	2394	<.05	22	−60	68	Superior parietal lobule
				8	−48	74	Postcentral gyrus
				4	−82	28	Cuneus
**Run 2, Placebo** **>** **Oxytocin**							
None							
**Mean of the runs, Oxytocin** **>** **Placebo**							
Left cerebellum, parahippocampal gyrus, hippocampus, fusiform gyrus, thalamus, lingual gyrus, visual cortex; right cuneus, visual cortex, cerebellum	Left	5348	<.05	−26	−32	−36	Cerebellum (culmen)
				−26	−48	20	White matter
				−30	−48	10	White matter
**Mean of the runs, Placebo** **>** **Oxytocin**							
None							

*k* number of voxels in the cluster, *p*_*FWE*_ FWE-corrected *p*-value

## Discussion

This is the first study to investigate the neurophysiological effects of oxytocin in CHR-P individuals. The key finding was that a single dose of intranasal oxytocin increased resting cerebral perfusion in the hippocampus, a region critically implicated in the pathophysiology of the CHR-P state and the later onset of psychosis. This finding is consistent with the only previous study of the effects of oxytocin on rCBF, which found left-lateralised increases in a large limbic cluster which included the hippocampus [27]. Our analysis of hippocampal subregions indicated that the largest effects of oxytocin were in the dentate gyrus and CA1 (although these analyses were exploratory and require confirmation and replication), while whole-brain analysis showed that oxytocin also modulated perfusion in the thalamus, parietal cortex and cerebellum.

Altered (increased) cerebral blood flow represents a core pathophysiological mechanism for psychosis onset [7–9] and is one of the few neuroimaging findings to have been replicated in independent CHR-P samples [8, 18, 19]. Increased hippocampal activity is also a key feature of preclinical models of psychosis [8, 12] and is thought to drive subcortical dopamine dysfunction [14]. Increases in hippocampal rCBF may therefore represent a disease-modifying target [7]. In view of this literature, our analyses focused on the hippocampal region. The left side (alone) was selected because previous CHR-P research has repeatedly implicated the left hippocampus in the pathophysiology of psychosis risk [7, 8, 10, 18, 20, 34, 35], with left (and not right) hippocampal blood flow associated with clinical outcomes (i.e., remission from a CHR-P state vs non-remission/transition to psychosis) [18]. In addition, previous oxytocin research has reported distinctly left-lateralised effects of oxytocin on cerebral blood flow [27].

We found that the effect of oxytocin on hippocampal rCBF in run 2 became non-significant after controlling for global signal. This may have reflected poorer signal-to-noise ratio in run 2 than run 1, as inspection of the raw data suggested there was greater variance in both hippocampal rCBF and global CBF values. Another possibility is that it was related to the much more pronounced effects of oxytocin on global rCBF during run 2 than in run 1. A final consideration is that our findings were influenced by the time course and dose-response effects of oxytocin, which may follow an inverted U-shaped curve [47, 48].

In exploratory analyses, we found that oxytocin increased rCBF in all of the hippocampal subregions that were examined, with the largest effects in the dentate gyrus and in CA1 (other subregions are discussed in the Supplementary Material). Previous neuroimaging research in CHR-P individuals suggests that CA1 is a key locus of dysfunction [8, 15]. The CA1 region plays an integral role in social and autobiographical memory [49] and CHR-P individuals show impairments in these domains [50]. In healthy individuals, oxytocin enhances social learning [51] and memory [24]. CA1 dysfunction is also at the centre of pathophysiological processes implicated in the onset of psychosis [7]; transition and/or non-remission from a CHR-P state is associated with enhanced CA1 perfusion and hypermetabolism [8, 18] and a gradual decline in CA1 volume [15]. Compared to other hippocampal subregions, CA1 has the highest number of GABAergic interneurons [15, 52] and an N-methyl-D-aspartate receptor (NMDAR) expression profile which confers enhanced susceptibility to glutamatergic alterations and excitotoxicity [53]—key features of the proposed neural circuit underlying psychosis onset [7]. In preclinical studies, oxytocin modulates this neural circuit by targeting GABAergic interneuron function and enhancing the signal-to-noise ratio of CA1 pyramidal cell firing [28, 29].

In terms of the dentate gyrus, CHR-P individuals whose symptoms had remitted were recently shown to have a longitudinal reduction in left hippocampal perfusion [18], which reference to a cytoarchitectonic atlas [44, 45] indicates had its peak coordinate in the left dentate gyrus. Another study reported reduced dentate gyrus volumes in CHR-P patients vs controls [17]. The dentate gyrus is thought to function as a computational pattern separator, with dysfunction here mechanistically linked to NMDAR hypofunction [54] and generation of spurious associations that may contribute to the onset of psychotic symptoms [55]. Patients with first-episode psychosis show deficits in pattern separation, which can be recreated in healthy volunteers using ketamine (NMDAR antagonist) challenge [54]. Interestingly, oxytocin is thought to exert its facilitatory effects on social recognition and behaviour via oxytocin receptors in the dentate gyrus, which recruit pattern separation circuits to minimise interference between similar social memories—at least in preclinical models [56]. In rats, activation of oxytocin receptors drives GABA release in the dentate gyrus in an action potential-dependent manner [57], and exogenous oxytocin has stimulatory effects on cell proliferation and adult neurogenesis, even under conditions of stress and elevated glucocorticoids, which also appears to be specific to the dentate gyrus [58]. These findings further demonstrate that oxytocin engages key pathophysiological circuits that are associated with the onset of psychosis.

We also investigated the effects of oxytocin at the whole-brain level. We found that oxytocin was associated with increased perfusion in large clusters spanning the hippocampus, parahippocampal gyrus and fusiform gyrus, as well as the cerebellum, and in run 2, the thalamus. Effects in these regions are consistent with previous work on (a) the effects of oxytocin on perfusion in healthy individuals [27], (b) high levels of oxytocin pathway gene expression and mRNA in the hippocampus, parahippocampal gyrus and thalamus (preprint [59]), and (c) the role of these regions in emotion processing and social cognition [21, 60, 61]. Increased perfusion was observed—albeit as part of a large cluster—in the left posterior hippocampus (including CA1 and dentate gyrus) at the whole-brain level across all runs, despite not surviving adjustment for global signal effects. The left-lateralised temporal lobe findings are in line with previous oxytocin work [27, 47, 60, 62] and predominantly left-hemisphere ROIs used in CHR-P neuroimaging studies [34, 63].

A separate healthy control group was not included in the current study because two previous independent CHR-P samples recruited from the same clinical service—the OASIS—have shown that hippocampal perfusion is altered in CHR-P individuals vs controls. These studies were large and the findings replicated, thus providing a priori evidence of hippocampal rCBF alterations in CHR-P individuals. Thus, we have used ROIs based on previous studies that reflect validated CHR-P vs control differences. However, future studies that include a parallel group of healthy volunteers would allow examination of the specificity and potential differential effects of oxytocin in CHR-P vs normative samples, as well as aiding the interpretation of the direction (increase vs decrease) of cerebral blood flow effects. Because we only tested one relatively mid-to-high range dose of oxytocin (40IU, which may be sufficient to cross-react with vasopressin receptors to give a vasopressin-like effect [64, 65]), we were not able to evaluate whether lower doses would show different effects (i.e., reduction of hippocampal perfusion). Given that previous studies have reported increased hippocampal perfusion in people at CHR-P [8, 18, 19], it may well be that a reduction in perfusion is the ultimate therapeutic target. These investigations were not possible in the current study, which was primarily an acute challenge to demonstrate disease-target engagement, but they provide the first evidence that intranasal oxytocin can alter cerebral blood flow in CHR-P individuals in target brain regions. Furthermore, while initial evidence of direct nose-to-brain transport has recently emerged [66], the exact mechanism by which it enters the brain is not fully understood, and differences in nasal anatomy and administration technique could influence the amount of oxytocin that reaches the brain. Our crossover (and counterbalanced order) design helped to control for this, but future research could use novel devices which may provide a more consistent and optimised delivery of oxytocin. Although none of the CHR-P participants were taking antipsychotic medication, a minority were taking antidepressants (*N* = 8) or benzodiazepines (*N* = 1), which could have affected the results. However, excluding these subjects did not alter the main results. We also excluded female subjects due to sexual dimorphism in oxytocinergic function [48, 60]. We did not include specific behavioural or symptom data because the study was designed (and therefore powered) to investigate the neurophysiological basis for the effects of oxytocin and to primarily show disease-target engagement. Finally, findings in CHR-P subjects can be influenced by sampling biases that modulate the level of risk for psychosis [67], but the level of risk in subjects from our local CHR-P clinic [36] has remained stable over recent years [68].

CHR-P individuals show deficits in social cognition [69] and altered neural responses during social and emotion processing fMRI tasks [70], which may contribute to a reduction in social and occupational functioning. Because oxytocin can have prosocial effects in healthy volunteers [23, 25] and in patients with schizophrenia [71], and modulates brain activation during social and emotion processing fMRI paradigms [72], this suggests that it might—subject to future research—be useful as a novel treatment in CHR-P subjects. However, while our results are promising in showing that oxytocin can engage brain regions strongly implicated in the onset of psychosis, they do not tell us about effects on symptoms, functioning, social cognition or any other CHR-P presentation, which limits the clinical interpretability of our findings. These outcomes remain important avenues for future research and we envisage that this study will provide the neurophysiological evidence in support of future longer-term clinical trials that can provide clinical validation. Furthermore, oxytocin has a good side effect profile; it is safe and well tolerated [73] and none of our participants reported adverse effects. At present, there are no licensed pharmacological treatments for this group, and although psychological interventions have been recommended, there is limited evidence that these are effective [2, 3]. Developing effective treatments for CHR-P subjects thus represents an unmet clinical need.

## Conclusions

The present study indicates that a single dose of oxytocin can significantly modulate hippocampal perfusion in people at CHR for psychosis. This suggests that oxytocin merits further investigation as a candidate novel treatment for this group.

## Funding and disclosure

This work was supported by the National Institute for Health Research (NIHR) Biomedical Research Centre (BRC) at South London and Maudsley NHS Foundation Trust and King’s College London (PFP, PM, DS); by a Brain & Behaviour Research Foundation NARSAD Award (grant number 22593 to PFP); and by the Department of Psychosis Studies, Institute of Psychiatry, Psychology & Neuroscience, King’s College London. DO is supported by the UK Medical Research Council (MR/N013700/1) and is a King’s College London member of the MRC Doctoral Training Partnership in Biomedical Sciences. The views expressed are those of the authors and not necessarily those of the NHS, the NIHR or the Department of Health and Social Care. The funders had no influence on the design, collection, analysis and interpretation of the data, writing of the report and decision to submit this article for publication. PFP has received advisory consultancy fees from Lundbeck outside of this work.

## Supplementary information

Supplementary Material
