# Supplementary material for: Oxytocin modulates hippocampal perfusion in people at clinical high risk for psychosis
Source: Neuropsychopharmacology. 2019 Jan 9;44(7):1300–9. doi: 10.1038/s41386-018-0311-6 (PMC6784972; doi:10.1038/s41386-018-0311-6)
Supplement: Supplementary file 1 — Supplementary Material [file 41386_2018_311_MOESM1_ESM.pdf]

OXYTOCIN MODULATES HIPPOCAMPAL PERFUSION IN PEOPLE AT CLINICAL HIGH RISK FOR PSYCHOSIS

Cathy Davies, Yannis Paloyelis, Grazia Rutigliano, Marco Cappucciati, Andrea De Micheli, Valentina Ramella-Cravaro, Umberto Provenzani, Mathilde Antoniades, Gemma Modinos, Dominic Oliver, Daniel Stahl, Silvia Murguia, Fernando Zelaya, Paul Allen, Sukhi Shergill, Paul Morrison, Steve Williams, David Taylor, Philip McGuire, Paolo Fusar-Poli

– SUPPLEMENTARY MATERIAL –

Methods

Design, materials, procedure

MRI acquisition parameters and procedures

Image processing

Statistical analysis

Results

Sensitivity analyses for primary results

Whole-brain results, adjusted for global effects

Additional Tables & Figures

Table S1. Whole-brain effects on CBF with adjustment for global effects

Figure S1. rCBF effects in left hippocampus without adjustment for global effects

Figure S2. rCBF effects in left hippocampal subregions without adjustment for global effects

Discussion

Whole-brain results adjusted for global signal effects

Further hippocampal subregions

References

Methods

Design, Materials, Procedure

Subjects were asked to abstain from using recreational drugs for at least one week prior to each MRI scan, and alcohol for at least 24 hours prior to each MRI scan. Urine screening was conducted before the scan for each participant.

The blinded spray bottles used for each session (containing oxytocin or matched placebo) were visually identical and dispensed by the Maudsley Hospital Pharmacy. Intranasal administration followed recommended guidelines [6] and a protocol adopted by a previous study conducted at our institute [7]. After a demonstration of the intranasal administration from a researcher using a spray bottle containing water, participants self-administered (in the presence of and with feedback from a researcher) one puff (4IU) of intranasal oxytocin (Syntocinon) or placebo every 30 seconds, alternating between nostrils, until 40IU (10 puffs) had been administered. The administration phase lasted approximately 4.5 minutes. A timer was started after the last puff had been administered and was used to coordinate the start of the first MRI sequence (see section below) run at 22 minutes post-administration (run 1), followed by the second run at 30 minutes (run 2).

Both the participants and researchers were blind to the (crossover) treatment sequence (AB or BA) allocation. A randomisation list was generated by the Maudsley Hospital Pharmacy, which determined whether a participant received oxytocin or placebo in their first study visit and vice versa for the second study visit. On recruitment of a study participant, an unblinded clinical trial pharmacist, who was not involved with the rest of the study, allocated the participant to one of the two sequences (AB, BA) based on the randomisation list. Allocation information was kept concealed in the Maudsley Hospital Pharmacy.

MRI acquisition parameters and procedures

All scans were conducted on a General Electric Discovery MR750 3 Tesla system (General Electric, Chicago, USA) at the Institute of Psychiatry, Psychology and Neuroscience, King's College London, using a 32-channel head coil. Measurement of Cerebral Blood Flow (CBF) was carried out using a 3D pseudo-continuous Arterial Spin Labeling (3D-pCASL) sequence during two consecutive runs, from 22-28 (run 1) and 30-36 (run 2) minutes post-intranasal administration.

Labelling of arterial blood was achieved with a 1525ms train of Hanning-shaped radio frequency pulses in the presence of a net magnetic field gradient along the flow direction

(the z-axis of the magnet). After a post-labelling delay of 2025ms, a whole brain volume was read using a 3D inter-leaved “stack-of-spirals” Fast Spin Echo readout [8], consisting of 8 interleaved spiral arms in the in-plane direction, with 512 points per spiral interleave. TE=11ms, TR=5135ms, and 56 slice-partitions of 3mm thickness were defined in the 3D readout. The in-plane FoV was 240×240mm. The spiral sampling of k-space was re-gridded to a rectangular matrix with an approximate in-plane resolution of 3.6mm. The sequence used 4 control-label pairs. CBF maps were computed from the mean perfusion weighted difference image derived from the four control-label pairs, by scaling the difference image against a proton density image acquired at the end of the sequence, using identical readout parameters. This computation was done according to the formula suggested in the recent ASL consensus article [9]. The sequence used four background suppression pulses to minimise static tissue signal at the time of image acquisition. The entire acquisition time of the 3D-pCASL sequence was 6:20 minutes for each run.

We also acquired a three-dimensional sagittal high-spatial-resolution Inversion Recovery Spoiled Gradient Echo (IR-SPGR) T1-weighted scan (TE=3.016ms, TR=7.31ms, TI=400ms, FoV=270mm). The final resolution of the image was 1.1 x 1.1 x 1.2mm.

Image Processing

ASL data were preprocessed using the Automatic Software for ASL Processing (ASAP) 2.0 toolbox [10] running in Statistical Parametric Mapping version 12 (SPM12; https://www.fil.ion.ucl.ac.uk/spm/) and Matlab R2017a using the following procedure [11,12] for each run: (1) the origin of CBF and 3D T1-weighted images was realigned; (2) 3D T1-weighted images were segmented to generate a binary mask including only brain tissues; (3) CBF maps were co-registered to the corresponding 3D T1-weighted images; (4) extra-cerebral signal was removed from the CBF map by multiplication of the “brain only” binary mask, obtained in step 2, with the CBF map in the space of the T1 image; and (5) T1-weighted scans and skull-stripped CBF maps were spatially normalised to MNI avg152 standard space. For each subject, a mean (average) CBF map was obtained from the two preprocessed CBF maps (runs 1 and 2) using the “imcalc” function in SPM12. Finally, CBF maps were spatially smoothed using a 6mm Gaussian smoothing kernel [11].

Statistical Analysis

Pre-scan anxiety scores

For pre-scan anxiety (STAI [13]) scores, across all subjects (N=29) combined, 4 individual items out of a total of 580 (20-item scale x 29 subjects) were missing. These 4 items were therefore imputed using next-observation-carried-backward [14].

Repeated-measured analyses of covariance (RM-ANCOVAs) in STATA

Because pre-scan anxiety scores and global grey matter CBF were scan-level covariates, the data were prepared in STATA in long format, which allows specification of a covariate value for each level of the repeated factor (drug: oxytocin vs placebo). This procedure allows controlling for time-varying covariates within a paired-samples (here within-subject) design.

Exploratory/supplemental analyses

For runs with significant results at the level of the whole hippocampus (run 1 only), we used analogous procedures to those described for the whole hippocampal ROI to extract mean rCBF values for each hippocampal subregion individually. Separate masks for left CA1, CA2, CA3, dentate gyrus, and subiculum were anatomically defined using the cytoarchitectonic probabilistic atlas [15] in the SPM Anatomy [16] toolbox (see Figure 3A in the main text). Mean rCBF values for each subregion ROI were extracted and log transformed due to deviations from distributional assumptions for parametric tests. These values were then entered into RM-ANCOVAs in STATA. No multiplicity correction was applied as subregion analyses were exploratory.

Results

Sensitivity analyses - hippocampal rCBF

Eight participants were taking antidepressants, one of which was also taking benzodiazepines. To ensure that inclusion of these cases was not unduly influencing our results, we repeated the primary analyses (left hippocampal ROI rCBF analyses) after exclusion of these 8 cases. Here, we found that compared to placebo, oxytocin administration was associated with increased hippocampal rCBF in run 1 ($F(1,19)=11.37$, $p=.0032$), run 2 ($F(1,19)=8.12$, $p=.010$) and the mean of runs ($F(1,19)=10.64$, $p=.0041$), all of which survived Hochberg multiplicity correction. After controlling for global signal effects, oxytocin administration was associated with increased hippocampal rCBF in run 1 ($F(1,18)=6.10$, $p=.024$), however, this result did not survive multiplicity correction. The effects were also no longer evident in run 2 ($F(1,18)=1.01$, $p=.33$) or in the mean of runs ($F(1,18)=3.68$, $p=.071$).

Whole-brain results, adjusted for global effects

Results adjusted for global effects are reported in Table S1 below. After controlling for global CBF effects, in run 1, oxytocin administration was associated with decreased perfusion in a cluster spanning the bilateral anterior cingulate and frontal cortices ($p_{FWE}<.05$), a separate cluster in the right inferior frontal gyrus ($p_{FWE}<.05$), and increased CBF in the left visual cortex ($p_{FWE}<.05$) (and see Discussion below). There were no suprathreshold whole-brain effects for either contrast in run 2 or in the mean of runs.

Tables & Figures

Table S1. Effects of oxytocin vs placebo on whole-brain CBF (with adjustment for global CBF effects)

Cluster Description	Hemis-phere	*k*	$P_{(FWE\text{-}corr)}$	Peak coordinates x	y	z	Peak Description
**Run 1, Oxytocin > Placebo**							
Left visual cortex, calcarine gyrus; right visual cortex	Left	620	<.05	-12	-72	16	Primary visual cortex (V1, V2)
				-6	-80	28	Visual association cortex (dorsal V3)
				-14	-72	-2	Visual association cortex (ventral V3)
**Run 1, Placebo > Oxytocin**							
Left anterior cingulate cortex/gyrus, frontal pole, superior medial frontal gyrus; bilateral mid orbital gyrus	Left	581	<.05	-2	36	26	Anterior cingulate cortex
				6	48	-12	Frontal pole
				-2	28	42	Superior medial frontal gyrus
Right inferior frontal gyrus	Right	383	<.05	50	14	22	Inferior frontal gyrus (pars opercularis)
				48	28	22	Inferior frontal gyrus (pars triangularis)
				60	4	12	Rolandic operculum
**Run 2, Oxytocin > Placebo**							
None							
**Run 2, Placebo > Oxytocin**							
None							
**Mean of runs, Oxytocin > Placebo**							
None							
**Mean of runs, Placebo > Oxytocin**							
None							

k, number of voxels in the cluster; P_{FWE}, FWE-corrected p-value.

Figure S1. rCBF Effects in Left Hippocampus. Bar charts showing mean hippocampal rCBF in the oxytocin and placebo conditions in runs 1 and 2 without adjustment for global effects.

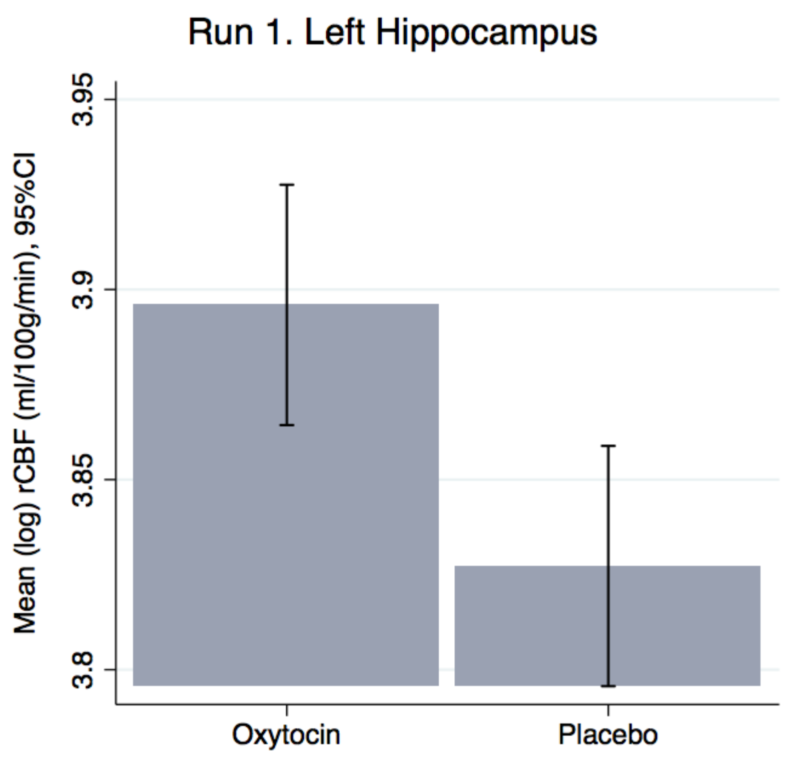

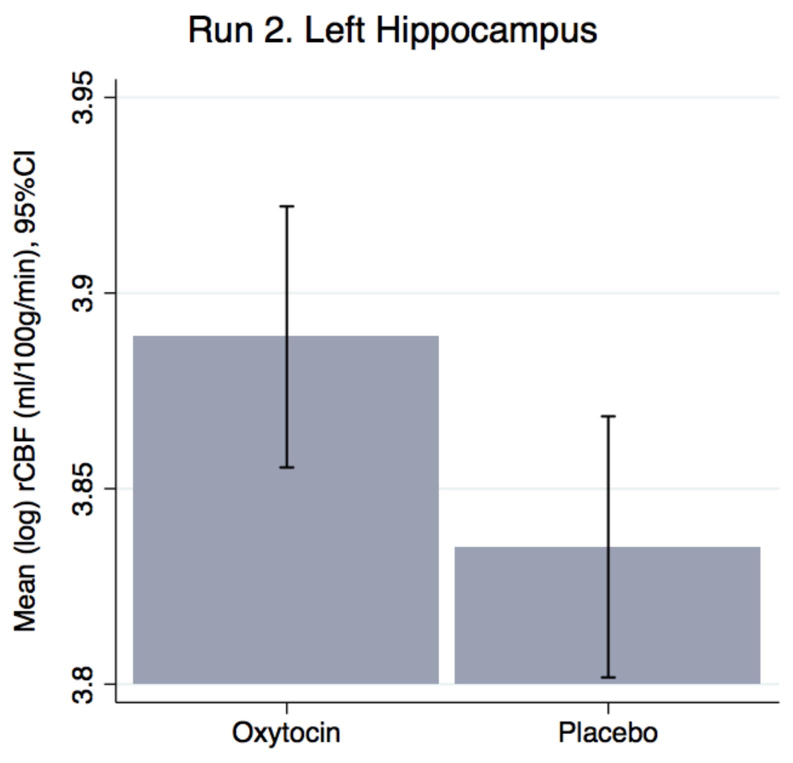

Figure S2. rCBF in Left Hippocampal Subregions. Bar charts showing mean hippocampal subregion rCBF in the oxytocin and placebo conditions in run 1 without adjustment for global effects.

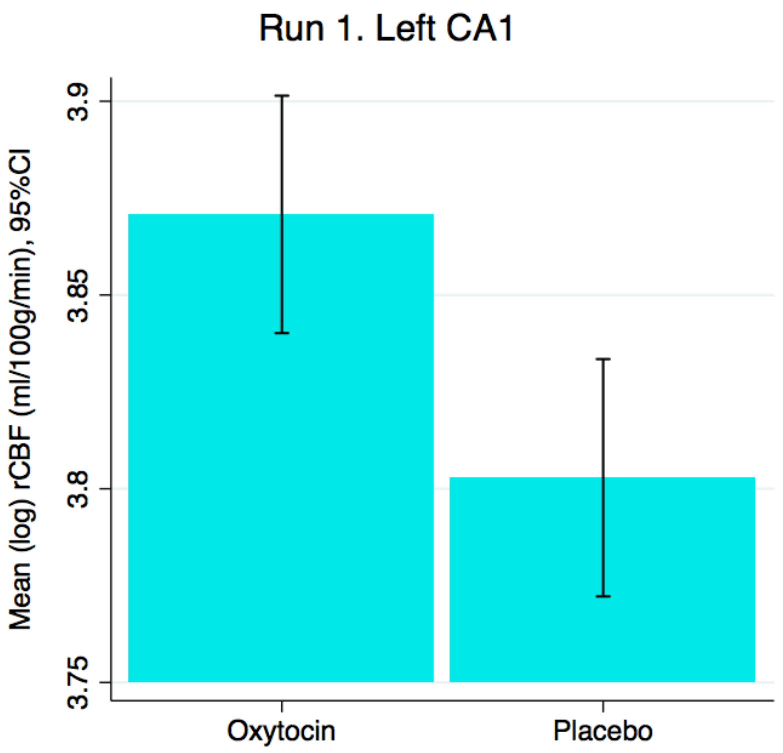

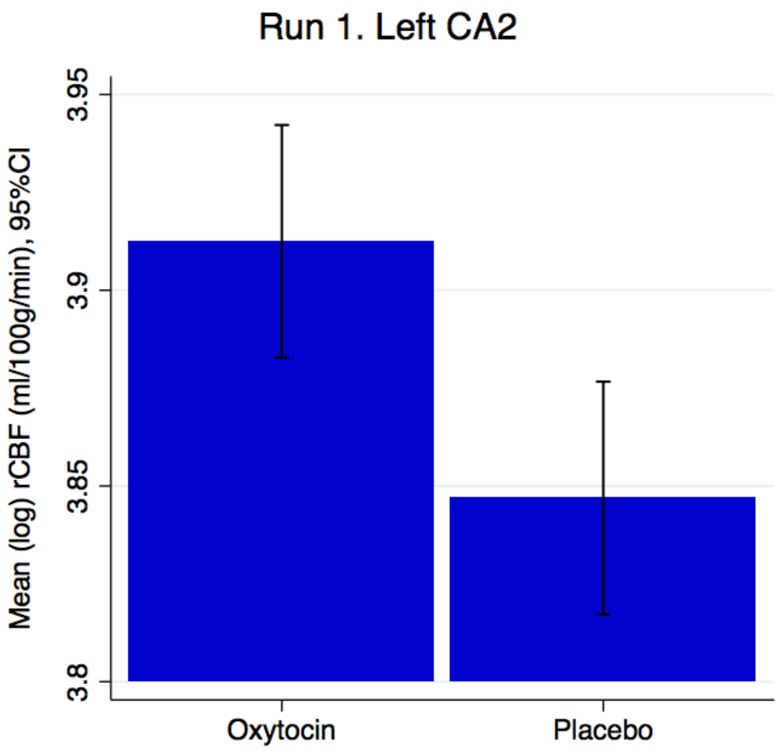

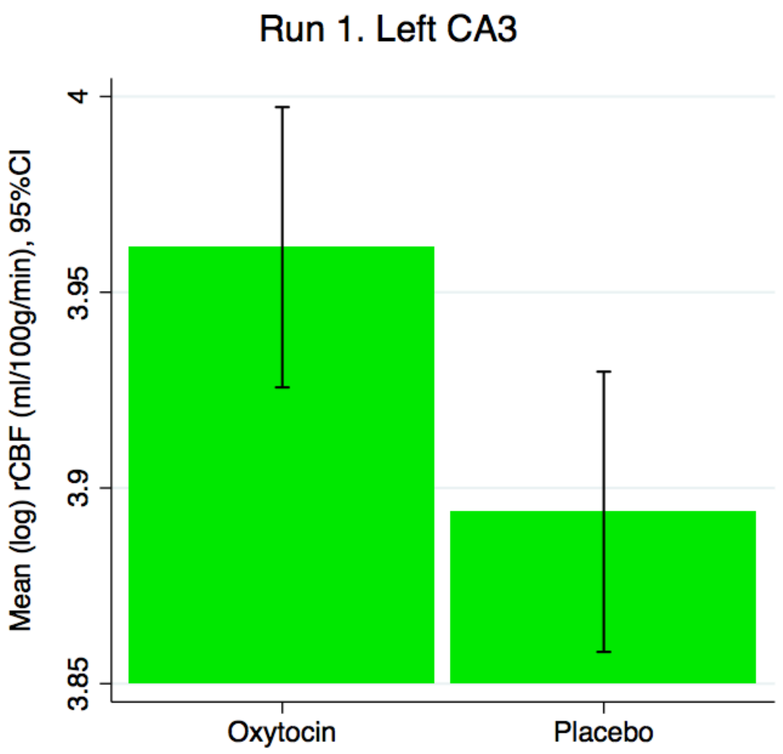
Run 1. Left CA3
Mean (log) rCBF (ml/100g/min), 95%CI
3.85
3.9
3.95
4
Oxytocin
Placebo

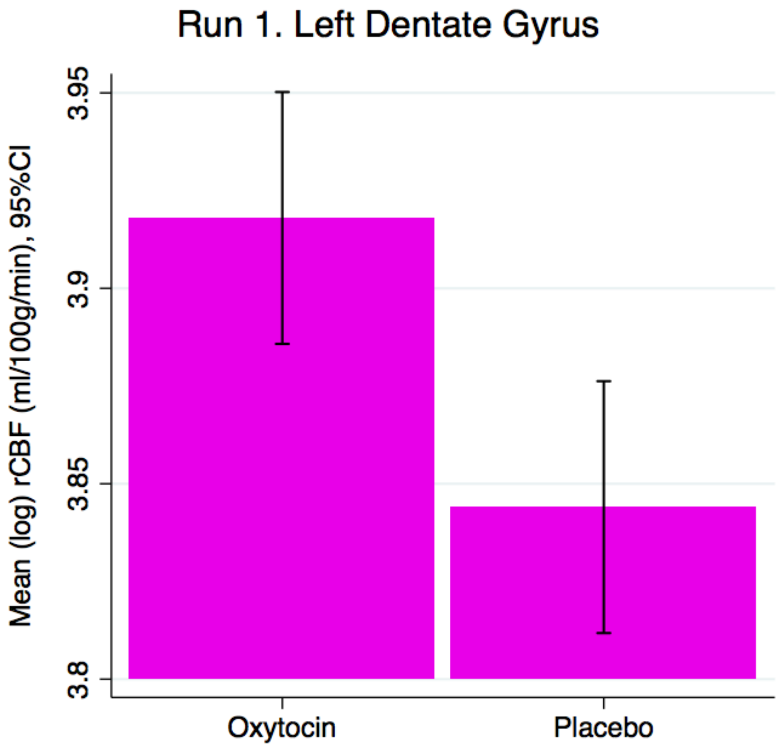
Run 1. Left Dentate Gyrus
Mean (log) rCBF (ml/100g/min), 95%CI
3.8
3.85
3.9
3.95
Oxytocin
Placebo

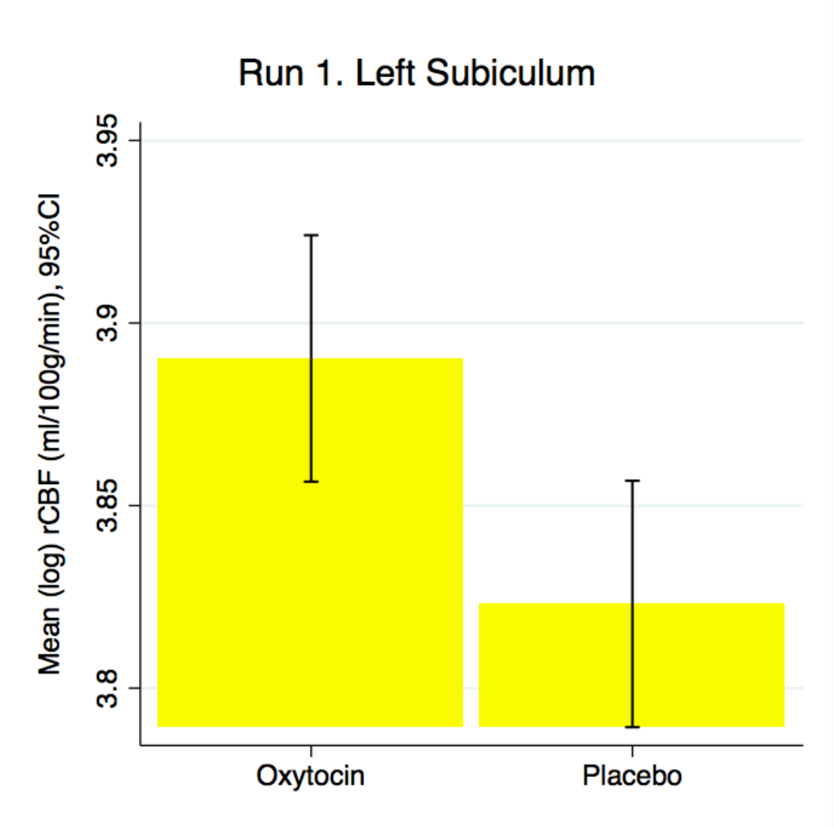
Run 1. Left Subiculum
Mean (log) rCBF (ml/100g/min), 95%CI
3.95
3.9
3.85
3.8
Oxytocin
Placebo

Discussion

Whole-brain results corrected for global signal effects

While the temporal/cerebellar whole-brain results (as reported in the main text) were not observed after inclusion of global signal as a nuisance covariate, there were a number of clusters that were significant, including increased perfusion in visual cortex, but perhaps more surprisingly, we also observed decreased perfusion across the anterior cingulate cortex (ACC) and inferior frontal gyrus (IFG) in the oxytocin vs placebo condition. Postmortem immunohistochemical studies have found oxytocin receptors and fibres in the ACC [17], and numerous associations between oxytocin receptor gene polymorphisms and ACC volume [18] as well as ACC/IFG functional activation have been reported [19,20]. Our findings are also consistent with the attenuated engagement observed in these regions in functional MRI studies [21] after oxytocin. However, this contrasts with the previous oxytocin study in healthy males, which found increased perfusion in the ACC and IFG in response to oxytocin [7], but it should be noted that this previous study [7] was a between-group comparison. These differential effects of oxytocin on the ACC/IFG might also be explained by differences in baseline rCBF in prefrontal regions in CHR-P individuals relative to controls, as have recently been reported [22].

Further hippocampal subregions

While we were *a priori* interested in CA1, which we have discussed along with the dentate gyrus in the main text discussion, we also found increased perfusion after oxytocin in the subiculum and CA2 subregion. As noted in the introduction, converging lines of evidence implicate CA1 hypermetabolism/hyperperfusion as a driver of the pathophysiological processes underlying the onset of psychosis [2]. However, research has shown that longitudinally, this CA1-originating dysfunction appears to progressively spread to the subiculum (and then to extra-hippocampal regions such as the frontal cortex) at the time of onset of psychosis in CHR-P individuals who transition vs those who do not [23]. This is combined with volumetric and morphometric shape changes occurring from the time of a CHR-P state to the onset of frank psychosis, again with the greatest changes observed in the subiculum (and CA1)[23]. Another study found selectively reduced subiculum volumes and worse verbal recall scores in familial high-risk individuals vs low genetic risk controls, with recall performance significantly correlated with subicular volume [24]. In terms of the CA2 subregion, studies have reported reduced CA2/3 volumes in non-remitted CHR-P individuals relative to healthy controls [25]. Reduced CA2/3 volumes [26,27] and greater longitudinal reductions in CA2/3 volume (which correlated with worsening general psychopathology scores over time) are also seen in patients with schizophrenia relative to healthy controls [28]. Intriguingly, oxytocin receptors are abundantly expressed in CA2 (at least in rodents) [29] and oxytocin receptor knock-out studies show that oxytocin signalling in CA2 is crucial for the persistence of long-term social recognition memory [30].

References

1. Lisman JE, Coyle JT, Green RW, Javitt DC, Benes FM, Heckers S, et al. Circuit-based framework for understanding neurotransmitter and risk gene interactions in schizophrenia. Trends Neurosci. 2008;31(5):234–42.
2. Lieberman JA, Girgis RR, Brucato G, Moore H, Provenzano F, Kegeles L, et al. Hippocampal dysfunction in the pathophysiology of schizophrenia: a selective review and hypothesis for early detection and intervention. Mol Psychiatry [Internet]. 2018 Aug 9;23(8):1764–72. Available from: http://www.nature.com/doifinder/10.1038/mp.2017.249
3. Krystal JH, Anticevic A, Yang GJ, Dragoi G, Driesen NR, Wang XJ, et al. Impaired Tuning of Neural Ensembles and the Pathophysiology of Schizophrenia: A Translational and Computational Neuroscience Perspective. Biol Psychiatry [Internet]. 2017;81(10):874–85. Available from: http://dx.doi.org/10.1016/j.biopsych.2017.01.004
4. Krystal JH, Anticevic A. Toward Illness Phase-Specific Pharmacotherapy for Schizophrenia. Biol Psychiatry [Internet]. 2015;78(11):738–40. Available from: http://dx.doi.org/10.1016/j.biopsych.2015.08.017
5. Modinos G, Allen P, Grace AA, McGuire P. Translating the MAM model of psychosis to humans. Trends Neurosci. 2015;38(3):129–38.
6. Guastella AJ, Hickie IB, McGuinness MM, Otis M, Woods EA, Disinger HM, et al. Recommendations for the standardisation of oxytocin nasal administration and guidelines for its reporting in human research. Psychoneuroendocrinology [Internet]. 2013;38(5):612–25. Available from: http://dx.doi.org/10.1016/j.psyneuen.2012.11.019
7. Paloyelis Y, Doyle OM, Zelaya FO, Maltezos S, Williams SC, Fotopoulou A, et al. A Spatiotemporal Profile of In Vivo Cerebral Blood Flow Changes Following Intranasal Oxytocin in Humans. Biol Psychiatry [Internet]. 2016;79(8):693–705. Available from: http://dx.doi.org/10.1016/j.biopsych.2014.10.005
8. Thedens DR, Irarrazaval P, Sachs TS, Meyer CH, Nishimura DG. Fast magnetic resonance coronary angiography with a three-dimensional stack of spirals trajectory. Magn Reson Med. 1999;
9. Alsop DC, Detre JA, Golay X, Günther M, Hendrikse J, Hernandez-Garcia L, et al. Recommended implementation of arterial spin-labeled Perfusion mri for clinical applications: A consensus of the ISMRM Perfusion Study group and the European consortium for ASL in dementia. Magn Reson Med. 2015;73(1):102–16.
10. Mato Abad V, García-Polo P, O’Daly O, Hernández-Tamames JA, Zelaya F. ASAP (Automatic Software for ASL Processing): A toolbox for processing Arterial Spin Labeling images. Magn Reson Imaging [Internet]. 2016;34(3):334–44. Available from: http://dx.doi.org/10.1016/j.mri.2015.11.002
11. Modinos G, Egerton A, McMullen K, McLaughlin A, Kumari V, Barker GJ, et al.

Increased resting perfusion of the hippocampus in high positive schizotypy: A pseudocontinuous arterial spin labeling study. Hum Brain Mapp [Internet]. 2018 Jun 8;(May):1–10. Available from: http://doi.wiley.com/10.1002/hbm.24231
12. McQueen G, Lally J, Collier T, Zelaya F, Lythgoe DJ, Barker GJ, et al. Effects of N-acetylcysteine on brain glutamate levels and resting perfusion in schizophrenia. Psychopharmacology (Berl) [Internet]. 2018 Aug 23;44:S81–2. Available from: http://ovidsp.ovid.com/ovidweb.cgi?T=JS&PAGE=reference&D=emexb&NEWS=N&AN=621900741
13. Spielberger C. Manual for the State-Trait Anxiety Inventory (STAI). Consult Psychol Press. 1983;
14. Higgins J, Green (editors) S. Cochrane Handbook for systematic reviews of interventions Version 5.1.0 [updated March 2011]. Cochrane Collab 2011. 2011;See 8.13.2.
15. Amunts K, Kedo O, Kindler M, Pieperhoff P, Mohlberg H, Shah NJ, et al. Cytoarchitectonic mapping of the human amygdala, hippocampal region and entorhinal cortex: Intersubject variability and probability maps. Anat Embryol (Berl). 2005;210(5–6):343–52.
16. Eickhoff SB, Stephan KE, Mohlberg H, Grefkes C, Fink GR, Amunts K, et al. A new SPM toolbox for combining probabilistic cytoarchitectonic maps and functional imaging data. Neuroimage. 2005;25(4):1325–35.
17. Boccia ML, Petrusz P, Suzuki K, Marson L, Pedersen CA. Immunohistochemical localization of oxytocin receptors in human brain. Neuroscience [Internet]. 2013;253:155–64. Available from: http://dx.doi.org/10.1016/j.neuroscience.2013.08.048
18. Furman DJ, Chen MC, Gotlib IH. Variant in oxytocin receptor gene is associated with amygdala volume. Psychoneuroendocrinology. 2011;36(6):891–7.
19. Loth E, Poline JB, Thyreau B, Jia T, Tao C, Lourdusamy A, et al. Oxytocin receptor genotype modulates ventral striatal activity to social cues and response to stressful life events. Biol Psychiatry. 2014;76(5):367–76.
20. Tost H, Kolachana B, Verchinski BA, Bilek E, Goldman AL, Mattay VS, et al. Neurogenetic effects of OXTR rs2254298 in the extended limbic system of healthy caucasian adults. Biol Psychiatry. 2011;70(9):37–9.
21. Wigton R, Radua J, Allen P, Averbeck B, Meyer-Lindenberg A, McGuire PK, et al. Neurophysiological effects of acute oxytocin administration: systematic review and meta-analysis of placebo-controlled imaging studies. J Psychiatry Neurosci [Internet]. 2015;40(1):E1-22. Available from: http://www.pubmedcentral.nih.gov/articlerender.fcgi?artid=4275335&tool=pmcentrez&rendertype=abstract

22. Kindler J, Schultze-Lutter F, Hauf M, Dierks T, Federspiel A, Walther S, et al. Increased Striatal and Reduced Prefrontal Cerebral Blood Flow in Clinical High Risk for Psychosis. Schizophr Bull. 2018;44(1):182–92.
23. Schobel SA, Chaudhury NH, Khan UA, Paniagua B, Styner MA, Asllani I, et al. Imaging Patients with Psychosis and a Mouse Model Establishes a Spreading Pattern of Hippocampal Dysfunction and Implicates Glutamate as a Driver. Neuron. 2013;78(1):81–93.
24. Francis AN, Seidman LJ, Tandon N, Shenton ME, Thermenos HW, Mesholam-Gately RI, et al. Reduced subicular subdivisions of the hippocampal formation and verbal declarative memory impairments in young relatives at risk for schizophrenia. Schizophr Res. 2013 Dec;151(1–3):154–7.
25. Ho NF, Holt DJ, Cheung M, Iglesias JE, Goh A, Wang M, et al. Progressive Decline in Hippocampal CA1 Volume in Individuals at Ultra-High-Risk for Psychosis Who Do Not Remit: Findings from the Longitudinal Youth at Risk Study. Neuropsychopharmacology [Internet]. 2017;42(6):1361–70. Available from: http://dx.doi.org/10.1038/npp.2017.5
26. Mathew I, Gardin TM, Tandon N, Eack S, Francis AN, Seidman LJ, et al. Medial temporal lobe structures and hippocampal subfields in psychotic disorders: Findings from the bipolar-schizophrenia network on intermediate phenotypes (B-SNIP) study. JAMA Psychiatry. 2014;71(7):769–77.
27. Haukvik UK, Westlye LT, Mørch-Johnsen L, Jørgensen KN, Lange EH, Dale AM, et al. In vivo hippocampal subfield volumes in schizophrenia and bipolar disorder. Biol Psychiatry [Internet]. 2015;77(6):581–8. Available from: http://dx.doi.org/10.1016/j.biopsych.2014.06.020
28. Ho NF, Iglesias JE, Sum MY, Kuswanto CN, Sitoh YY, De Souza J, et al. Progression from selective to general involvement of hippocampal subfields in schizophrenia. Mol Psychiatry. 2017;22(1):142–52.
29. Mitre M, Marlin BJ, Schiavo JK, Morina E, Norden SE, Hackett TA, et al. A Distributed Network for Social Cognition Enriched for Oxytocin Receptors. J Neurosci [Internet]. 2016;36(8):2517–35. Available from: http://www.ncbi.nlm.nih.gov/pubmed/26911697%5Cnhttp://www.pubmedcentral.nih.gov/articlerender.fcgi?artid=PMC4764667
30. Lin Y-T, Hsieh T-Y, Tsai T-C, Chen C-C, Huang C-C, Hsu K-S. Conditional Deletion of Hippocampal CA2/CA3a Oxytocin Receptors Impairs the Persistence of Long-Term Social Recognition Memory in Mice. J Neurosci [Internet]. 2017;38(5):1896–17. Available from: http://www.jneurosci.org/lookup/doi/10.1523/JNEUROSCI.1896-17.2017
